# Summary of Twenty-First Century Great Conversations in Art, Neuroscience and Related Therapeutics

**DOI:** 10.3389/fpsyg.2018.01428

**Published:** 2018-08-08

**Authors:** Juliet L. King

**Affiliations:** Indiana University, Purdue University Indianapolis, Indianapolis, IN, United States

**Keywords:** art, neuroscience, creative arts therapies, art therapy, mobile brain body imaging, neuroaesthetics

## Abstract

Transdisciplinary collaboration is the future of knowledge making in advanced post-industrial societies and there is a growing awareness that the most vexing problems we face cannot be solved by any single discipline. Best practices for complex and challenging physical and mental disorders require a multi-disciplinary approach, yet there is a void in bridging the gap between the most contemporary models. It is in this capacity that the Twenty-First Century Great Conversations in Art, Neuroscience, and Related Therapeutics serves as a missing link. It was with active minds and a collective spirit that artists, scientists, therapists, physicians, engineers, technology experts, healthcare practitioners, and researchers from across the globe transcended historical silos to explore the capacities for collaborative partnerships to influence the health of patients and the amelioration of disease. Hosted at Indiana University-Purdue University Indianapolis (IUPUI), presenters shared insights through didactic sessions and panel discussions aligned with three tracks led by prominent experts in their respective fields: (1) Neuroaesthetics, Anjan Chatterjee, MD; (2) Creativity and Consciousness, Arne Dietrich, PhD; and (3) Mobile Brain/Body Imaging (MoBI), Klaus Gramann, PhD. The goals for this symposium were developed from a vision which embraces cross-disciplinary intersectionality, a merging of viewpoints, and active dialogue surrounding the development of a common language with which to advance the Creative Arts Therapies and neurosciences. The goal was also to contribute to the development of a simplified roadmap to enhance and enrich the CATs with a greater understanding of neuroscience and the available technologies that can assist in research.

Transdisciplinary collaboration is the future of knowledge making in advanced post-industrial societies and there is a growing awareness that the most vexing problems we face cannot be solved by any single discipline. Current best practices for complex and challenging physical and mental disorders require a multi-disciplinary approach, yet there remains a void in bridging the gap between the most contemporary models. It is in this capacity that the *Twenty-First Century Great Conversations in Art, Neuroscience and Related Therapeutics* serves as a missing link. This international symposium was hosted at Indiana University-Purdue University Indianapolis (IUPUI), where the schools of Art, Medicine, Engineering, Informatics, Health, and Rehabilitation Sciences, Nursing and Liberal Arts joined healthcare practitioners and researchers from across the globe to transcend historical silos and explore the capacities for collaborative partnerships to influence the health of patients and the amelioration of disease.

The symposium took place over 3 days and was attended by more than 120 people from 10 countries. 13 speakers shared insights through didactic sessions and panel discussions aligned with three tracks led by prominent experts in their respective fields: (1) Neuroaesthetics, Anjan Chatterjee, MD; (2) Creativity and Consciousness, Arne Dietrich, PhD; and (3) Mobile Brain/Body Imaging (MoBI), Klaus Gramann, PhD. Although there are many conferences that focus on the intersection of arts and sciences, the goals for this symposium were specifically developed from a vision which embraces cross-disciplinary intersectionality, a merging of viewpoints, and active dialogue surrounding the development of a common language with which to advance the Creative Arts Therapies (CATs) and neurosciences. The goal was also to contribute to the development of a simplified roadmap to enhance and enrich the CATs with a greater understanding of neuroscience and the available technologies that can assist in research. *(See* *Appendix* *A* *for Speaker Topics and Primary Themes and* *Appendix* *A* *for recommended readings based on Keynote Addresses)* (Supplementary Material). This perspective article is written through the lens of the symposium organizer, an art therapist, and is intended to highlight common themes extracted from the three keynote addresses and offer commentary for how these themes can be translated into research potentials at the intersection of our respective disciplines.

CATs embrace the variances of subjective artistic expression and its value in representing more completely the psyche of the individual, while neuroscientists typically strive for precise data acquisition in advancing the understanding of brain structures and functions. CATs rely upon the creative process and non-verbal symbolic expression as contributing factors for effective intervention and are positioned to understand that rigor in a scientific experiment that cultivates data inclusive of generalizability is just as important as arts-based research that calls upon intuition and phenomenological inquiry to inform what it is that we are seeking to understand. Creative Arts Therapists and neuroscientists need to evolve existing common language that will allow for communication and connection across disciplines and cultural barriers. Often times our seemingly distinct fields use the same words with different meanings which challenges the ability to communicate and may complicate our discourse. For example, “bottom up” and “top down” processing means something completely different to an engineer, a neurologist and an art therapist.

Through the identification of specific research questions that utilize a common scientific language, the CATs have greater capacities to provide insight into the links between cognitive, affective, and symbolic expression and brain function. Simultaneously, the translation of computational neuroscience data and neural correlates to human behavior is an expansive and rich terrain upon which the CATs have enormous potential to contribute. With a comprehensive understanding of the neurological mechanisms involved in creative expression, Creative Arts Therapists have more power to advance and perfect such forms of therapy, establish proof of what works and what does not and create models for delivering optimal treatments to better serve our patients.

This summary serves to disseminate the primary elements from the three Keynote Addresses and panel dialogue of these *Great Conversations*, offer integrative commentary for how the material translates to the research of the Creative Arts Therapist, and set the stage for future collaborative work in the coming years. The privilege to design and organize this symposium would not be possible without the support and guidance of Dr. Robert Pascuzzi, Chairman of the Department of Neurology for the IU School of Medicine. Many thanks for the input and participation of art therapist and research assistant Kaitlin Knapp in the design and implementation of the symposium and the preparation of this manuscript. Special thanks to planning committee members Alexandra Shaikh, JD Hall, MC Jill Ditmire, filmmaker Leigh DeNoon, and the graduate art therapy students that helped with the conference proceedings. Much gratitude is extended to the Indiana University New Frontiers in Arts and Humanities, the Indiana Clinical Translational Sciences Institute, IU School of Medicine Department of Neurology, Herron School of Art and Design, Efroymson Family Fund, and the Buckingham Foundation for the financial support for this event. ^*^note: permission was obtained to report the names and content of participants.

## Neuroaesthetics

### Anjan chatterjee, MD

Neuroaesthetics is a branch of empirical aesthetics that uses neuroscience to understand aesthetic experiences at the neurological level. Dr. Anjan Chatterjee called upon the work of Gustav Fechner to explain the origins of neuroaesthetics and described how properties of the world are systematically related to properties of the mind. There is an outer psychophysics, which relates properties of the world and mind, and an inner psychophysics, in which properties of the brain, of the nervous system, relate to properties of the mind.

There is a cognitive neuroscience of aesthetics and a cognitive neuroscience of art that are often related, but not identical. For example, one can have aesthetic experiences of natural objects such as faces and landscapes and also abstract objects like mathematics. Mathematicians talk about beautiful theorems and elegant proofs, and aesthetic experiences can occur when things are removed from art. It would be a mistake to think that aesthetic processes, either perception or production, occur in one part of the brain. This idea is categorically wrong. Dr. Dietrich expanded on this in his Keynote Address with what he referred to as the *brief and frightening reign of the right hemisphere*: Creativity is not localized and although hemispheric specialization is of heavy interest to a neuroscientist, and there are many cognitive functions that show this laterization effect, creativity is not one of them.

Aesthetic experiences are among the most complex of brain functions. The brain sorts different pieces of the world (stimuli) into different modules that carry out specialized processing. Some of these modules classify objects like faces and bodies and body movements. It appears that these same modules also evaluate these objects and likely work in concert with the brains reward systems to produce our emotional responses regardless of whether they are delight or disgust (Chatterjee, 2016). For example, when people are looking at attractive faces, parts of the visual cortex that are specialized in processing faces tends to be active. Simultaneously, the reward systems that are in the front of and deep in the brain are active. (Including the orbitofrontal cortex, dorsal medial prefrontal cortex, nucleus accumbens, and the insula). The general system for valuation and rewards seem to be activated by attractive faces. Dr. Chatterjee explained that the cortical systems in the human brain interact with the deeper systems and provide a context in which we approach our *wants* and enjoy our *likes*.

#### Commentary

Understanding more thoroughly the connections between visual information processing and reward systems provides ample opportunity to study the nature of creative expression in clinical treatment with the addictions population. Creative Arts Therapists can significantly contribute to these inquiries by helping to translate what the science of the brain might look like in a clinical context. Can we compare an attractive face to a drug? Can we compare non-invasive stimuli with a stimulant drug and affect the reward system? If we know that we change our brain chemistry when we engage in activity, how can we learn more about the differences that making art and viewing art have on the reward systems? On neurotransmitters? Recent studies in physiology of creative expression and aesthetic experiences help to conceptualize the many ways of initiating research in this arena (Kaimal et al., 2017a; Pelowski et al., 2017). Contributing to the research on addictions by testing art therapy intervention through a neuroaesthetic lens is timely, given the current opioid crisis in our nation and overall prevalence of addictions and substance abuse.

Building upon great strides in the field of art therapy (Walker et al., 2016, 2018), a clinical population ripe with potential for collaborative inquiry is brain injury. One reason for this is that it may be easier to assess a change in a resting or task-negative state pre and post CAT intervention since a brain injury tends to be more static in nature compared to a condition such as Post Traumatic Stress. Chatterjee and Coslett (Chatterjee and Coslett, 2014) affirms that brain damage can alter the available parts of the brain dedicated to the overall artistic output that becomes the product of a different coordination of components. He makes an analogy where we might think about neural systems like a suspended mobile, which rests on the equilibrium of its weights. If one of the weights is removed, the entire structure could collapse, yet also find itself in a new resting state. Similarly, brain damage may render the artist incapable of continuing the work or may create a new equilibrium where the artistic production shows alternative configurations. Creative Arts Therapists observe how creative expression in the context of the therapeutic relationship promotes the capacity for the brain to balance itself into a homeostasis like a Calder mobile. Pairing the metaphor with the science allows for a common language to explore these phenomena more thoroughly and make specific links between disciplines. We might observe the neuroplastic pathways of creative expression more closely by looking at the compensatory functions found through artistic expression following a brain injury.

Although more difficult to study due to the nature of neurodegenerative disease, at the *Great Conversations*, Dr. Chatterjee mentioned that Alzheimer's Disease (AD) is an area of inquiry in desperate need for scientific data to show efficacy of therapeutic intervention. Funding opportunities for AD should provide motivation for all therapeutic disciplines to generate sound hypotheses that test existing models of treatment and although there is solid research available, it is notoriously difficult to obtain quantifiable data in treatment of AD through the Creative Arts Therapies (Cowl and Gaugler, 2014). The CATs rely heavily on the engagement of imaginative systems in the production of symbolic expression, and the ability to bypass language and access less conscious material while attending to task is an important, if not crucial aspect of treatment that needs more attention.

Creative Arts Therapists readily see transformation in clinical practice by observing patient engagement in artistic expression that often results in the capacity to form narrative around images that otherwise would and could not be articulated. This is often accompanied by a reduction in symptoms and behavioral change. The Creative Arts Therapist often witnesses that many people who have endured brain injury develop new artistic talent post injury. Creative Arts Therapists are trained with awareness that creating in solitude is different than in the context of another. If the Creative Arts Therapist was trained to understand more completely the neurological mechanisms of aesthetic expression there would be an invigorated opportunity to develop specific and verifiable (and falsifiable) hypotheses that would support clinical observations with proof. Simultaneously, Creative Arts Therapists are positioned to explore more thoroughly what and who is being treated beyond a cluster of symptoms and without relying solely on diagnoses and brain science to define the person.

Although not a popular figure in neuroaesthetics, it is important to mention Sigmund Freud here. Freud was not focused exclusively on anatomical localization but he was invested in the energy transfer of the dynamic unconscious. Creative Arts Therapists have long relied upon theories of psychoanalysis and call upon Freud to explain a synthesis that occurs through conscious and unconscious expression when symbolized through art process and product. This is the native tongue of the Creative Arts Therapist. It is exciting to consider here the work of Kandel (1998) who articulates a biological approach to psychiatry through an integrated perspective that emerges from Freudian theories and might promote a *renaissance* of psychoanalytic thought (p. 11). Neuropsychologist Zaidel (2016) emphasizes imaging research that shows how unconscious and conscious cognitive systems interact in our perception of artwork at the neural level. Neuropsychology is crucial in the advancement of our knowledge of cognitive processing systems and helps make the quantitative shifts necessary to more completely understand the role of neuroaesthetics (Chatterjee and Coslett, 2014). By studying the brains response to aesthetic stimuli we learn more about the interactive conscious and unconscious systems, which gets us steps closer to validating with science what is referred to as symbolic and nonverbal communication.

Neuroaesthetics does not currently address therapeutic implications and further investigation of how the physiological and psychological aspects of aesthetic experience relate to one another is an important goal for the future (Chatterjee et al., 2010). The knowledge of the scientist is enhanced with the clinical knowledge of the therapist who specializes in artistic self-expression to facilitate behavioral change and symptom reduction. Dr. Girija Kaimal, art therapy researcher and conference attendee, stated that “Art therapists are well positioned to identify behaviors, patterns of visual self-expression, clinical profiles of specific populations and interpersonal dynamics. As Creative Arts Therapists are not traditionally trained in neuroscience, measurement tools or technology, they are well positioned to partner with those who are. This will help the work of neuroscientists to become grounded in clinical practice while also serving to advance knowledge in both fields.” (G. Kaimal, personal communication, 2017).

## Creativity and consciousness

### Arne dietrich, PhD

Looking for creativity in the brain is Sisyphus' work! Among many topics, Dr. Arne Dietrich focused on key themes to help the audience understand the true nature of creativity in the brain. He began by elucidating a fallacy of belief called the *divergent thinking paradigm*, which states that if both divergent and convergent thinking lead to creative thinking then there is a problem because we do not yet know what it is about divergent thinking that is creative. Creativity in terms of divergent thinking is a compound construct; it is complex and there are many cognitive functions involved. For the mechanistically-minded neuroscientist, divergent thinking becomes a beast, said Dr. Dietrich, as it is too amorphous and too large to tackle. We do not know what neural or cognitive processes, and to what extent, go into divergent thinking to make it measurable with neuroimaging. We can measure working memory, perceptual processes, categorization, and attention processes with functional MRI, but we cannot at this time measure divergent thinking, nor is there a neural signature for complex psychological constructs.

We have a tendency to think about creativity as one thing—it is not one particular trade or characteristic, but rather the plurality of processes that can come in a variety of shapes, forms and sizes. You cannot isolate what you are studying with the creative process; if you cannot isolate the topic because you have false category information combined with a compound construct, you can't decipher what an MRI shows because you haven't isolated the mechanisms. Neuroscientists look for mechanisms and in order to identify them, they need to delineate the processes that the mechanisms occur within.

We have mechanisms that occur inside the brain that do not map very well with what we experience. The best way to understand this is by considering a computer—when we drag and click something into the trash it is simple on the user-friendly surface, but the computer is undergoing a much more complicated series of events. Creative Arts Therapists observe a multidimensionality of symbol formation through nonverbal expression found in imagery, music and movement, and like the neuroscientist, would benefit from distilling creativity into small enough pieces so that each piece can be tested as a part of a larger component. To do this will generate greater evidence for why the creative process is considered an integral and life enhancing component of CATs. Dr. Dietrich affirmed that researchers would also benefit from breaking down creativity into types. There is no such thing as a simple overarching creativity process, mechanism, or brain localization. Rather, there are different types, processes and anatomical features that are opposing. Based on current knowledge in neuroscience and evolutionary theory these are the deliberate mode, the spontaneous mode, and the flow mode, all of which are different in terms of neuroanatomical features and processes. All types of creativity, however, are multifaceted and completely embedded in the brain according to cognitive neuroscience.

#### Commentary

Although challenging, the articulation of a cogent definition of creativity is a useful goal that can enhance innovative collaborations and inform cross-disciplinary research. Dr. Klaus Gramann asserted in his Keynote Address on Mobile Brain/Body Imaging (MoBI) that in order to understand what happens in the brain, we must understand what happens when we move. Motion requires efficacy and nothing costs as many neural resources as movement. The flow mode of creativity requires motion and movement and engages implicit processing, the basal ganglia and the limbic system. Art therapists have questioned whether some behavior states are more connected to flow than others (Chilton, 2013). Creative Arts Therapists who work with neurological conditions such as Parkinson's Disease and Movement Disorders can contribute to a more thorough understanding of what is happening in the brain throughout the recovery of the disease state by documenting behavior change and symptom management through both verbal and nonverbal artistic expression. If the motor system of the brain is damaged then the quality of skill may be different and these behavioral variances contribute rich information that informs the questions to explore mechanisms of creativity in the brain.

Tremendous strides have been made to develop cogent theories of art therapy assessment and intervention through the use of the Expressive Therapies Continuum (ETC) (Hinz, 2009; Lusebrink, 2014; Lusebrink and Hinz, 2016). This theoretical model is based upon the assumption that media properties evoke different levels of visual information processing. Dr. Hinz (2014) documented several pertinent research questions that are guided by the ETC and include: *What differential experiences are evoked by the basic media used in art therapy?* Based on what we now know about neuroscience, it would serve the CAT researchers to start backwards and focus on a single thing we can prove. For example, does the brain produce a distinctive response to specific aesthetic stimuli at the neurological level? If we were able to identify that the use of media evoked a different neurological response in an individual then we would have more evidence to support and test hypotheses related to the ETC. Wearable Electroencephalography (EEG) technology such as MoBI would allow for an understanding of what types of media elicit what types of brainwave activity and may illuminate where the activation takes place. This data would contribute to the growing knowledge of what happens in the brain when we “*art*,” and would also provide a scientific framework for media choice during art therapy intervention.

Dr. Sandra Gaskell, a clinical speech pathologist who is in the process of obtaining credentials as an art therapist and psychologist attended the symposium and shared her insights for viable avenues of interdisciplinary research. Dr. Gaskell is interested in assessment and suggested that a primary challenge with using the ETC as an assessment is that we are not yet able to validate any scoring mechanism for it, as is the case for most art therapy assessments. She suggests that if we were able to map the brain during an art therapy assessment we might be able to identify the neural activations that take place throughout the procedure and potentially correlate these with elements of the assessment. Further, if we distill symptom clusters for medical and psychiatric illnesses, apply an art therapy intervention and test changes in brain wave activity with EEG we may be able to isolate what brain activities take place and identify what can be improved. Speech pathologists specialize in understanding communication and the pathological variants of conditions such as Selective Mutism, Aphasia, and TBI. Creating research protocols with experts in nonverbal communication based upon existing models is both logical and pertinent.

Art therapists understand that the *Creative level* of the ETC is the optimal state where psychic integration takes place yet seem to refer to the healing potentials of creativity without fully recognizing how unwieldy the term really is to neuroscientists like Dr. Dietrich. It would benefit both CATs and neurosciences to explore with more clarity about what we mean by creativity. One way to do this might be to “localize the lesion,” so to speak. In neurology, “lesion studies” are an established method of breaking down parts and connections to see how the brain is operating with imaging technology. Lesions in humans with injuries and diseases are natural experiments and in animals they are planned and controlled experiments that help us to clarify mechanisms, circuits and interconnections. How can art therapists distil components of the creative process so that we can speak with more scientific certainty about how and why our interventions actually work? If we study a distinction between a deliberate type of creativity and a flow state based on client engagement at different levels of the ETC, how might this help to clarify the value of the CATs as a profession that offers evidence-based interventions? With the advancement of neuroimaging technologies like Mobile Brain/Body Imaging, we now have greater capacities to “take our investigations into the wild.” (K. Gramann, personal communication, 2017).

## Mobile brain/body imaging (MoBI)

### Klaus gramann, Phd

At the core of MoBI is the understanding that cognition is deeply rooted in the body's interaction with the world and happens in a dynamically changing environment (Wilson, 2002). Movement through and physical interaction with the environment alters our cognition, and consequently the brain dynamics that accompany cognitive processes are also likely to change (consider here Dr. Chatterjee's comments on inner and outer psychophysics). If we leave behind the restrictions of traditional brain imaging approaches we can investigate different behavioral states and how they change the brain dynamic state. Traditional assessment tools for brain function such as EEG do not allow for movement of participants because they are too stationary, the brain signals become contaminated with movement-induced artifact (the “noise” that gets in the way of an EEG reading), and ultimately this results in a reduction of the behavior dimensionality that we seek to assess.

MoBI was developed in 2007 with the idea that cognition and brain dynamics are embodied, and the natural cognition that makes use of physical structure—that which allows, uses and incorporates movement—feeds back into cognition itself (Makeig et al., 2009). If we know that movement changes cognitive processes, then we have more ability to understand the underlying neurological dynamics. MoBI identifies three factors: cognition, brain dynamics and movement, and explores their interdependency by recording all dimensions in synchrony through the use of technology such as EEG and fNIRS (Functional Near Infrared Spectroscopy). Although complex, we have a general understanding that MoBI provides the ability to analyze data while people actively behave in space. Art such as sculpture provides us with information for how human perception is perceived in the three dimensions. When we compare sculpture to a 2-D painting we obtain data for what information our body gives us when we move around compared to a stationary view of art. MoBI is relatively low cost and provides opportunities to conduct research while engaging in a task of creativity and artistic expression.

#### Commentary

The use of MoBI is particularly relevant for the CATs, as movement is inherent in artistic expression through visual art, music, and movement. For example, Dance Movement Therapists work in the integrative space of mind and body connection and help clients regulate by engaging the nervous system through kinesthetic activity. MoBI opens wide the exploration of how a Dance Movement Therapy intervention can improve the physical symptoms of an illness by comparing tractable brain activity with observed behavioral change through a rating instrument. This is significant to working with trauma. It is now accepted that the brain does not integrate sensory experiences easily after trauma and that traumatic memories are stored in our bodies and in areas of the brain that we have less conscious access to. Gramann (personal communication, 2017) emphasizes it is possible that learning new sensory-motor associations when experiencing the same sensory input but associating this with a different output can help overwrite traumatic memories, which can be measured and tested with this innovative technology and contribute to providing scientific evidence for working models of therapeutic intervention.

Art therapist Linda Chapman, who writes prolifically on the neurobiology of trauma, urges us to consider the virtually untapped application of the visual system in art therapy and how this interacts with our bodies. Through the use of the visual system it is possible to address challenges such as phantom limb pain and paralysis. For example, stroke victims who have paralysis on one side might sit in front of a mirror and use the hand and arm without paralysis in front of the mirror. The brain is “tricked” into thinking that it sees the other limb, which opens up the neural pathways so the person can use the arm with former paralysis. Although most of the innovative research in this area is in the medical profession (Chan et al., 2007; Mercier and Sirigu, 2009), this is a rich area of discovery for those interested in medical art therapy (L. Chapman, personal communication, 2018). Similar to the prior examples, MoBI technology can assist in the identification of neurological mechanisms that make this physical and behavioral change possible.

There are several possibilities for how a neurological change may emerge, starting with structural changes and ending with functional changes (keeping in mind that structural changes can impact functional changes). Most structural changes in the brain require CT or MRI to visualize. Functional changes can be assessed and quantified with EEG and functional imaging. Functional EEG changes might be expressed in: (1) changes in the frequency domain (e.g., less alpha attenuation after intervention); (2) changes in the time domain (e.g., faster onset of a component or reduced amplitudes); (3) changes in connectivity (e.g., from parietal hubness to an increase of connecting activity in other areas).

Studying functional connectivity within parts of the brain is logical, the investigations of which may rest upon what has been done so far in neuroimaging and art therapy (Belkofer and Konopka, 2008; Belkofer et al., 2014; Kruk et al., 2014; King et al., 2017). Capitalizing on the framework set forth it makes sense to expand these investigations through the use of technology that can be utilized in the active and engaged artmaking state, the investigations of which have also been initiated (Kaimal et al., 2016, 2017a,b). Exploring more thoroughly established clinical areas that are proven to be effective with the non-verbal therapies, or that are deficient in specific clinical symptoms that are observed, is an area of rich opportunity for transdisciplinary research for Creative Arts Therapists to use MoBI. From here we could seek to establish standardized tests that indicate a specific deficit or ability and compare the EEG measures in such standardized tests pre and post. Working to distil the correlations between brain function and creative expression and then applying in clinical trials is well within the reach of the Creative Arts Therapies and neuroscience research and will significantly contribute to the advancement of both fields.

Dr. Gramann provided rich information on a valuable tool for approaching research in the Creative Arts Therapies. As with the other Keynote speakers, he cautioned the audience on the limits of technology, the capacities of the neuroscientist to inform therapeutics, and the precision with which we need to proceed in order to be successful when attempting to integrate art, neuroscience and related therapeutics in both theory and research.

## Conclusion

Outcomes are necessary to record the value of collaboration and this paper serves as the first published deliverable of the *Twenty-First Century Great Conversations*. This symposium was a thought provoking, inspiring and collegial experience that showcased the courageous potential to let go of ego and embrace different yet equally valuable perspectives. By removing the silos of our respective disciplines, we have the (action) potential to generate new connections and pathways of thinking; we are embodied creativity.

## Author contributions

The author confirms being the sole contributor of this work and approved it for publication.

### Conflict of interest statement

The author declares that the research was conducted in the absence of any commercial or financial relationships that could be construed as a potential conflict of interest.
