# Supplementary material for: Summary of Twenty-First Century Great Conversations in Art, Neuroscience and Related Therapeutics
Source: Front Psychol. 2018 Aug 8;9:1428. doi: 10.3389/fpsyg.2018.01428 (PMC6099956; doi:10.3389/fpsyg.2018.01428)
Supplement: Supplementary file 1 [file Data_Sheet_1.pdf]

Appendix A - Speaker Topics and Primary Themes

**Topic**	**Speaker**	**Primary Theme**
Neuroaesthetics	Anjan Chatterjee, MD	(see text)
Neurodegenerative Disease and Creative Art Therapies	Robert Pascuzzi, MD	The necessity of a range of therapeutics for patient and caregiver and call for research that uses conventional scientific methods to establish proof.
Empathy in Medical Student Education	Emily Beckman, MA, DMH	The use of narrative and storytelling to develop moral imagination and create empathy in medical learning
Art Therapy and Neuroscience	Juliet King, MA, ATR-BC, LPC, LMHC	The continuum of scientific inquiry and how art, therapies and neuroscience research enhance all disciplines
Art Therapy and Continuous Traumatic Stress	Myra Saad, MA, ATR	The distinction of continuous traumatic stress from other types of trauma and the importance of a humanitarian approach to art therapy centered in the continuity principle.
Neuroscience of Creativity and Consciousness	Arne Dietrich, PhD	(see text)
Arts and Humanities in Medicine and Education	Jeffrey Rothenberg, MD, MS	The value of visual strategies to hone thinking skills and the integration of art to encourage empathic and humanistic healers
Epilepsy Self-Management	Janice Buelow, RN, PhD, FAAN	The use of art therapy to improve the communicative capacities for people who have epilepsy.
Dance Movement Therapy	Heidi Fledderjohn, MA, BC-DMT, RYT	How Dance Movement Therapy engages the nervous system and the use of the body to translate and communicate our emotional states and interaction with the environment.
Art as Pilgrimage and Sanctuary	Jawshing Arthur Liou, MFA	Art making as a medium for distancing the source of tragedy and providing a means to confront it.
Design for Healing Environment/Art Design for Civic Practice	Michael Kaufmann, MLIS	How to create space to support better health outcomes and to facilitate a social infrastructure that can transcend barriers of language and culture.
Mobile Brain-Body Imaging	Klaus Gramann, PhD	(see text)
Psychological Distress and Dissociation	Petr Bob, PhD	Correlations between Freudian theories and contemporary neurobiological understanding of stress, traumatic memory and dissociation.
Music Therapy: Music Based Interventions	Debra Burns, PhD, MT-BC	How the music therapist uses both external evidence from research studies and internal understanding of the meaning of music to create interventions for patients.

Informatics: The Science and Art of Gameplay	Travis Faas, MS	The use of Virtual Reality in gameplay and the use of EEG and GSR to generate data through human interaction with the games themselves.
Neuroscience, Multi-Modal Imaging and Creative Arts Therapies	Lukasz Konopka, PhD	The brain as an oscillator; the use of EEG and technology to evaluate patient vulnerabilities and recommend more specific forms of therapeutic intervention which includes the Creative Arts Therapies.
