# Supplementary material for: Summary of Twenty-First Century Great Conversations in Art, Neuroscience and Related Therapeutics
Source: Front Psychol. 2018 Aug 8;9:1428. doi: 10.3389/fpsyg.2018.01428 (PMC6099956; doi:10.3389/fpsyg.2018.01428)
Supplement: Supplementary file 2 [file Data_Sheet_2.pdf]

Appendix B: Recommended References

**Topic**	**Citation**
Neuroaesthetics	Pearce, M. T., Zaidel, D. W., Vartanian, O., Skov, M., Leder, H., Chatterjee, A., Nadal, M. (2016). Neuroaesthetics: The cognitive neuroscience of aesthetic experience. *Perspectives on Psychological Science, 11*(2), 265-279.
	Chatterjee, A., & Vartanian, O. (2016). Neuroscience of aesthetics. *New York Academy of Science, 1369*. 172-194.
	Chatterjee, A. (2015). The neuropsychology of visual art. In J. P. Huston, M. Nadal, F. Mora, L. F. Agnati, & C. J. Cela-Conde (Eds.), *Art aesthetics, and the brain* (341-356). Oxford: Oxford University Press.
	Chatterjee, A., & Vartanian, O. (2014). Neuroaesthetics. *Trends In Cognitive Sciences, 18*(7), 370-375.
	Chatterjee, A. (2004). The neuropsychology of visual artistic production *Neuropsychology*, *41*(11), 1568-1582.
Creativity and Consciousness	Dietrich, A. (2004). The cognitive neuroscience of creativity. *Psychonomic Bulletin and Review, 11*, 1011-1026.
	Dietrich, A. (2007). *Introduction to consciousness: Neuroscience, cognitive science, and philosophy*. New York, NY: Palgrave MacMillan.
	Dietrich, A., & Kanso, R. (2010). A review of EEG, ERP, and neuroimaging studies of creativity and insight. *Psychological Bulletin, 136*(5), 822-848.
	Dietrich, A. (2015). *How creativity happens in the brain.* New York, NY: Palgrave MacMillan.
	Dietrich, A., & Haider, H. (2015). Human creativity, evolutionary algorithms, and predictive representations: The mechanics of thought trials. *Psychonomic Bulletin & Review, 22*, 897-915.
Mobile Brain-Body Imaging	Jungnickel, Evelyn & Gramann, Klaus. (2016). Mobile Brain/Body Imaging (MoBI) of Physical Interaction with Dynamically Moving Objects. *Frontiers in Human Neuroscience*. 10. 10.3389/fnhum.2016.00306.
	Gramann, K., Ferris, D.P., Gwin, J., Makeig, S. (2014). Imagining natural cognition. International Journal of Psychophysiology: Official Journal of the International Organization of Psychophysiology. 91: 22-9. PMID
	Gramann, K., Jung, T. P., Ferris, D. P., Lin, C. T., & Makeig, S. (2014). Towards a new cognitive neuroscience: modeling natural brain dynamics. *Frontiers E-books.*
	Gramann, K., Gwin, J. T., Bigdely-Shamlo, N., Ferris, D. P., & Makeig, S. (2010). Visual evoked responses during standing and walking. *Frontiers in human neuroscience*, *4*, 202.
	Makeig, S., Gramann, K., Jung, T-P, Sejnowski, TJ, Poizner, H. (2009). Linking brain, mind and behavior: The promise of mobile brain/body imaging (MoBI). *International Journal of Psychophysiology*, 73(2)
